# Chidamide and Radiotherapy Synergistically Induce Cell Apoptosis and Suppress Tumor Growth and Cancer Stemness by Regulating the MiR-375-EIF4G3 Axis in Lung Squamous Cell Carcinomas

**DOI:** 10.1155/2021/4936207

**Published:** 2021-06-11

**Authors:** Xu Huang, Nan Bi, Jingbo Wang, Hua Ren, Desi Pan, Xianping Lu, Luhua Wang

**Affiliations:** ^1^Department of Radiation Oncology, National Cancer Center, Cancer Hospital, Chinese Academy of Medical Sciences and Peking Union Medical College, Beijing 100021, China; ^2^Department of Radiation Oncology, Harbin Medical University Cancer Hospital, Harbin, Heilongjiang 150040, China; ^3^Shenzhen Chipscreen Biosciences, Shenzhen 518057, China; ^4^Department of Radiation Oncology, National Cancer Center, Cancer Hospital & Shenzhen Hospital, Chinese Academy of Medical Sciences and Peking Union Medical College, Shenzhen 518000, China

## Abstract

As a selective histone deacetylase (HDAC) inhibitor developed in China, chidamide has been applied for the treatment of refractory peripheral T-cell lymphoma (PTCL) and multiple solid tumors, including lung cancer. However, the underlying mechanisms are not well elucidated. In our present study, we found that chidamide and radiation acted synergistically to suppress cell and xenograft growth of lung squamous cell carcinoma cells by inducing cell apoptosis. Moreover, chidamide alone or a combination of chidamide and radiation treatment inhibited cancer cell stemness. miRNA microarray analysis demonstrated that miR-375 was the highest upregulated microRNA (miRNA) in NCI-2170 and NCI-H226 cells treated with chidamide alone or treated with chidamide plus radiation, compared with normal control. Inhibition of miR-375 attenuated the promoting effect of chidamide alone and chidamide plus radiation-induced NCI-2170 and NCI-H226 cell apoptosis and reverted the suppression of cancer stemness caused by chidamide alone or chidamide plus radiation treatment. Moreover, EIF4G3, a scaffold protein in the translation initiation complex, was found to be a direct target of miR-375 based on the luciferase reporter assay and western blot analysis. Interestingly, both chidamide alone and chidamide plus radiation treatments suppressed the mRNA and protein expression of EIF4G3. Silence of EIF4G3 also induced cell apoptosis and suppressed tumor growth in NCI-2170 and NCI-H226 cells. These data suggest that chidamide shows a synergistic effect with radiation therapy on lung squamous cell carcinomas by modulating the miR-375-EIF4G3 axis, which may afford an effective strategy to overcome the drug resistance of chidamide in clinical cancer therapy.

## 1. Introduction

Lung cancer is one of the leading causes of cancer-associated deaths all over the world, with a high metastatic potential [1]. Lung cancer can be divided into two main groups: small-cell lung cancers (SCLCs) and non-small-cell lung cancers (NSCLCs) [2]. NSCLCs account for 85% of lung cancer and can be further classified into four subtypes according to their histological and molecular features: lung large-cell carcinomas (LCLCs), lung neuroendocrine tumors (LungNETs), lung adenocarcinomas (LUADs), and lung squamous cell carcinomas (LSCCs) [3]. LSCCs are the main type of NSCLC showing strong malignancy. Although advanced treatment strategies and technologies such as surgical treatment, radiotherapy, and chemotherapy have been developed rapidly, the five-year survival rate among patients with LSCC remains very poor with increased risk of recurrence [4]. Therefore, it is urgently needed to furtherly understand the molecular mechanisms underlying LSCC initiation and progression and to seek effective methods for early detection and treatment.

Chidamide, a selective inhibitor of HDAC1, 2, 3, and 10 developed wholly in China, has been entered into clinical trials both in the United States and China. In December 2014, chidamide has been approved by the China Food and Drug Administration (CFDA) as a treatment strategy for peripheral T-cell lymphoma (PTCL) [5]. Interestingly, accumulating studies have demonstrated that chidamide shows an effective antitumor activity in multiple solid tumors, including liver cancer, colon carcinoma, and lung cancer [6–11]. In lung cancer, Hu et al. have performed a phase I trial of chidamide combined with paclitaxel and carboplatin in patients with advanced NSCLC and found that a combination treatment of chidamide and paclitaxel or carboplatin was tolerated without unanticipated toxicities or pharmacokinetic interactions [6]. Chidamide has also been reported to enhance the suppressive effect of platinum on NSCLCs [11]. However, the underlying mechanisms through which chidamide suppresses lung cancer are unclear.

MicroRNAs (miRNAs) represent a class of noncoding short RNAs with 19–24 nucleotides in length, which was highly conserved in eukaryotes. miRNAs play an important role in regulating multiple physiological and pathological processes by binding to the 3′-untranslated regions (3′-UTRs) of target genes [12, 13]. Dysregulation of miRNA has been implicated in the initiation and progression of a wide range of cancers, including liver, gastric, breast, lung, and colorectal cancers. For instance, Tian et al. found that silence of miR-203 promotes tumor cell growth and invasion by upregulating the SNAI2 in prostate cancer [14]. miR-22 inhibits breast cancer cell proliferation and increases paclitaxel sensitivity by suppressing N-RAS [15]. Aberrant expression of miRNAs has also been observed in lung cancer. For example, downregulation of miR-98-5p in NSCLC suppresses NSCLC proliferation and metastasis by targeting TGFBR1 [16]. miR-5195-3p inhibits cell proliferation, migration, and invasion in human NSCLC by targeting MYO6 [17].

Among these cancer-related miRNAs, miR-375 was initially identified as a critical regulator of insulin secretion and a novel therapeutic target for diabetes treatment [18]. Further studies have demonstrated that miR-375 participates in various cancer types by targeting several critical target genes including ATG7, AEG-1, YAP1, SP1, IGF1R, JAK2, and PDK1 [19]. The deregulation of miR-375 in tumors can be caused by a variety of mechanisms such as aberrant promoter methylation [20–22]. Deregulation of miR-375 can also be used as a biomarker for cancer prediction and diagnosis [23, 24]. In lung cancer, Jin et al. have reported that miR-375 expression was obviously increased in lung adenocarcinoma and SCLCs but reduced in LSCCs [25]. However, the exact role of miR-375 in lung cancer, especially in LSCCs, is not fully understood.

In the present study, we found that a combination of chidamide and radiation treatment promoted synergistic cytotoxicity and suppressed tumor stemness in LSCCs. Importantly, miR-375 was upregulated in NCI-2170 and NCI-H226 cells treated with chidamide alone or with chidamide plus radiation, compared with normal control. In addition, suppression of miR-375 attenuated chidamide alone and chidamide plus radiation-induced NCI-2170 and NCI-H226 cell apoptosis and suppressed tumor growth and stemness. Moreover, EIF4G3 was identified as a direct target of miR-375. Interestingly, both chidamide alone and chidamide plus radiation treatments suppressed the mRNA and protein expression of EIF4G3. Silence of EIF4G3 also induced cell apoptosis and suppressed tumor growth in NCI-2170 and NCI-H226 cells. These data suggest that chidamide shows a synergistic effect with radiation therapy on lung squamous cell carcinomas by modulating the miR-375-EIF4G3 axis.

## 2. Materials and Methods

### 2.1. Cell Cultures and Treatment

Human lung squamous cell carcinoma NCI-H2170 and NCI-H226 cells were obtained from ATCC (Manassas, VA, USA) and maintained in DMEM (Invitrogen, Carlsbad, CA, USA) with 10% fetal bovine serum (FBS; GIBCO, Waltham, MA, USA) and 1% penicillin/streptomycin (Beyotime, Shanghai, China) at 37°C in a 5% CO_2_ incubator. Chidamide (BioVision, Milpitas, CA, USA) was diluted in dimethyl sulfoxide (DMSO; Sigma-Aldrich, Shanghai, China). Cells were exposed to 300 nM of chidamide for 24 h or/and 6 MV X-ray radiation using a linear accelerator (Elekta; Stockholm, Sweden) at single doses of 0, 1, 2, and 4 Gy.

### 2.2. miRNA Mimic, Inhibitor, and siRNA Transfection

Cells were cultured to about 75% confluence before transfection. Control mimic, miR-375 mimic, control siRNA, and EIF4G3 siRNA were transfected into cells using Lipofectamine RNAiMAX Reagent (Thermo Fisher Scientific, Waltham, MA, USA) following the manufacturer's manual. Forty-eight hours after transfection, the cells were applied for the following experiment. The siRNA oligos were synthesized by Santa Cruz (CA, USA).

### 2.3. Cell Proliferation

Cell Counting Kit-8 was applied to determine the proliferation rate of NCI-H2170 and NCI-H226 cells with indicated treatment as previously described. Briefly, two thousand treated cells were plated into 96-well plates. Then, CCK-8 solution was added at the harvest time and incubated for an additional 30 min. The absorbance was determined at 450 nm on the microplate reader (Molecular Devices, Walpole, MA, USA).

### 2.4. Apoptosis Assay

Apoptosis assay was performed by Annexin V/PI double staining using the Annexin V-FITC apoptosis detection kit (BD Biosciences, Pharmingen, CA, USA) following the standard manual. Briefly, treated cells were washed with cold 1x PBS and resuspended in 1x binding buffer. Five *μ*l of FITC-labeled Annexin V and 5 *μ*l of propidium iodide (PI) were added to the suspended cells and gently mixed, following by the incubation at room temperature for 15 min in dark. At last, the samples were detected on the flow cytometer (BD Biosciences).

### 2.5. miRNA Microarray

miRNA microarray was performed as previously described [26].

### 2.6. Real-Time RT-PCR

Real-time RT-PCR was performed as previously described [27]. The primers used for real-time PCR detection were as follows: 5′-GTCGTATCCAGTGCAGGGTCCGAGGTATTCGCACTGGATACGACGGTTTG-3′ (miR-375 reverse transcription), 5′-GTGCAGGGTCCGAGGT-3′ (miR-375, forward), 5′-GCGCGACGAGCCCCTCGCT-3′ (miR-375, reverse), 5′-CTCGCTTCGGCAGCACA-3′ (U6, forward), 5′-AACGCTTCACGAATTTGCGT-3′ (U6, reverse), 5′-CCACAGCGCCATGTTGGAT-3′ (EIF4G3, forward), 5′-GATCTTTATCCCCCTCCCCG-3′ (EIF4G3, reverse), 5′-GCACCGTCAAGGCTGAGAAC-3′ (GAPDH, forward), and 5′-ATGGTGGTGAAGACGCCAGT-3′ (GAPDH, reverse).

### 2.7. Luciferase Reporter Assay

The luciferase reporter assay was determined using the psi-CHECK2 dual-luciferase system (Promega, Madison, WI, USA) following the standard manual. The QuickMutation™ Site-Directed Mutagenesis Kit (Beyotime, Shanghai, China) was applied for construction of EIF4G3 3′-UTR reporter plasmids with a mutant miR-375 binding site. Primer sequences used for construction of these plasmids were as follows: 5′-GCGCGATCGCAACTTCAAATACACAAAATG-3′ (EIF4G3-WT, forward), 5′-GCGTTTAAACCTGTCCAAAGGAGAAGTCAC-3′ (EIF4G3-WT, reverse); 5′-AGGCTTGTAAATACATACTTGTTTTATTTAAAAAAAC-3′ (EIF4G3-Mut1, forward), 5′-GTTTTTTTAAATAAAACAAGTATGTATTTACAAGCCT-3′ (EIF4G3-Mut1, reverse); 5′- CACTTTGAAAATATAAACTTGTTTTAAAGACAAAC-3′ (EIF4G3-Mut2, forward), and 5′-GTTTGTCTTTAAAACAAGTTTATATTTTCAAAGTG-3′ (EIF4G3-Mut2, reverse).

### 2.8. Western Blot Analysis to Determine Protein Expression

Western blot analysis was performed as previously described [28]. The primary antibodies were as follows: anti-EIF4G3 (AV40487; Sigma, Shanghai, China), anti-Bax (ab182733; Abcam), anti-BCL2 (#2872; Cell Signaling Technology), and anti-*β*-actin (AF0003; Beyotime).

### 2.9. ALDEFLUOR Assay and Flow Cytometry

The ALDEFLUOR Kit (Stemcell Technologies) was used for ALDH^+^ cell analyses according to the manufacturer's manual. For each sample, one-half of cells was treated with 50 mM of diethylaminobenzaldehyde (DEAB) to define negative gates.

### 2.10. Sphere Formation Assay

Sphere formation assay was determined as previously described [29].

### 2.11. Xenograft Mouse Model

Treated cells were subcutaneously injected into both sides of flank areas of 6–8-week-old BALB/c nude mice for 42 days. Tumor volumes were measured using the following equation: 0.5 × length × width^2^ each other day after palpable tumors appeared. The study protocol was approved by the Animal Care and Use committee of Harbin Medical University.

### 2.12. Immunohistochemistry

Immunohistochemistry assay was performed as previously described [30].

### 2.13. Statistical Analysis

All data were expressed as mean ± standard deviation (SD). Statistical analysis was performed using the software GraphPad Prism 5. Student's *t*-test was used to determine the statistical differences, in which *p* < 0.05 was considered to be significant.

## 3. Results

### 3.1. Both Chidamide Alone and Chidamide Plus Radiation Combinational Treatment Synergistically Promote Cell Apoptosis and Suppressed Cancer Cell Stemness in NCI-H2170 and NCI-226 Cells

**Figure 1:**
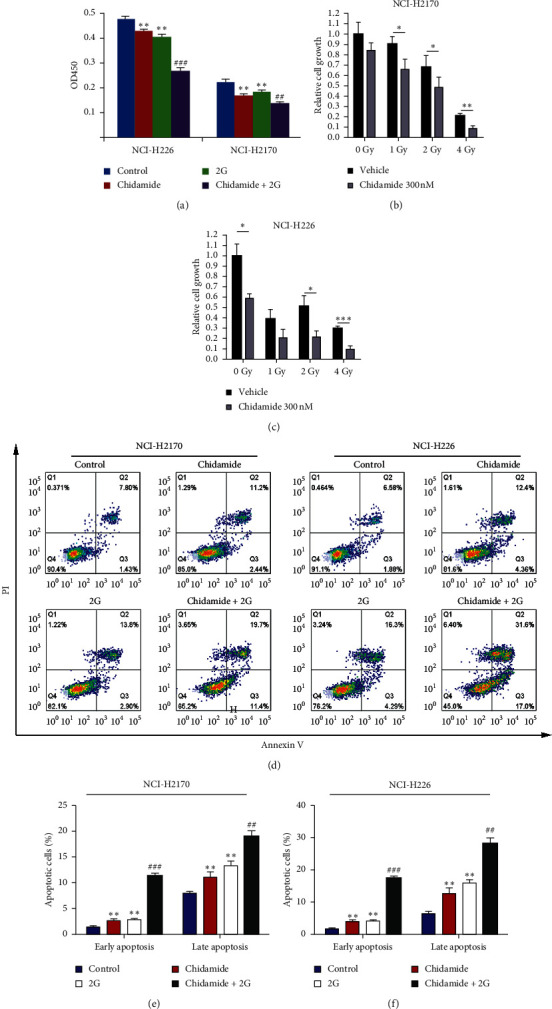
Chidamide or chidamide combined with radiation synergistically promotes apoptosis in NCI-H2170 and NCI-H226 cells. (a) CCK8 assay to determine the cell viability of NCI-H2170 and NCI-H226 cells with chidamide (300 nM), radiation (2 Gy), and their combinational treatment, respectively. (b-c) The proliferation rate of NCI-H2170 (b) and NCI-H226 (c) cells with chidamide (300 nM) combined with radiation treatment at single doses of 0, 1, 2, and 4 Gy. (d)–(f) Annexin V-PI double staining to determine the apoptosis rate of NCI-H2170 and NCI-H226 cells with chidamide (300 nM), radiation (2 Gy), and their combinational treatment.

Initially, to determine the effect of chidamide on cellular proliferation in LSCC cells, we treated NCI-H2170 and NCI-226 cells with 300 nM of chidamide for 24 h or/and 6 mV X-ray radiation using a linear accelerator at single doses of 0, 1, 2, and 4 Gy. The results indicated that chidamide, radiotherapy, and their combinational treatment inhibited cell proliferation in NCI-H2170 and NCI-H226 cells (Figures 1(a)–1(c)). Next, to explore the possible mechanism underlying chidamide-regulating proliferation of LSCC cells, we intended to testify whether cellular apoptosis could be contributed to the synergistic anticancer effect of chidamide and radiation on LSCC. After treating the NCI-H2170 and NCI-226 cells with 300 nM of chidamide for 24 h and/or with 2 Gy radiation, the effect of chidamide, radiotherapy, and their combinational treatment on cell apoptosis was determined by flow cytometry. Both the early and late apoptosis rates were significantly increased in cells treated with chidamide, radiotherapy, and their combination (Figures 1(d)–1(f)). The results demonstrate that chidamide and radiation synergistically inhibit LSCC cell proliferation potentially via inducing cellular apoptosis.

**Figure 2:**
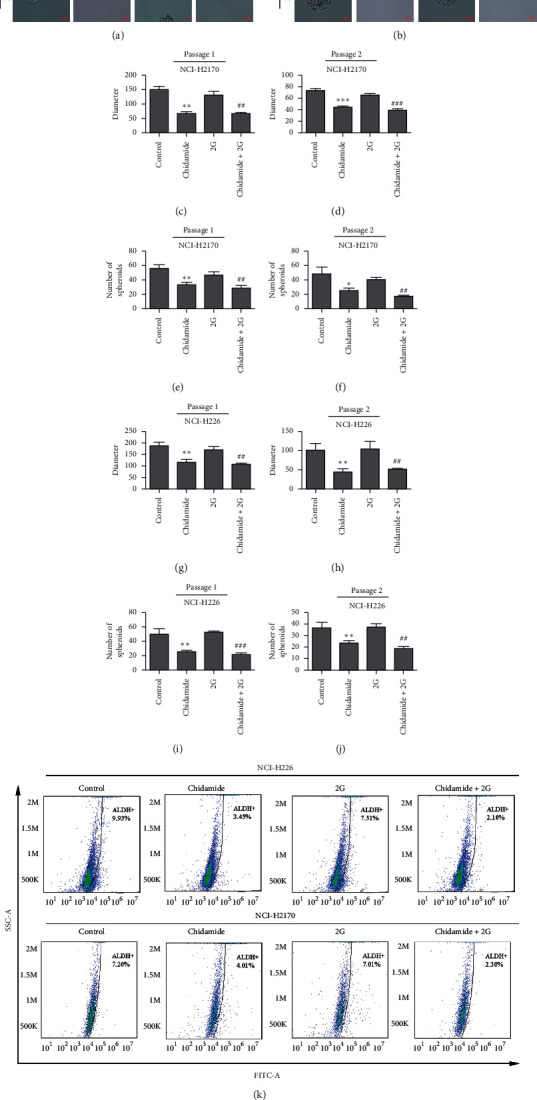
Chidamide or chidamide combined with radiation treatment suppressed cancer stemness in NCI-H2170 and NCI-H226 cells. (a–j) Sphere formation assay to determine the self-renewal capacities of NCI-H2170 and NCI-H226 cells treated with chidamide alone or a combination of chidamide and radiation. The sphere sizes and number were calculated in Figures 2(c)–2(j). (k–m) Treated cells with chidamide alone (300 nM) or combined with radiation treatment were subjected to ALDEFLUOR assay and the population of ALDH^+^ cells were counted by flow cytometry. Statistical significance was determined by Student's *t*-test and indicated by ^*∗*^*p* < 0.05; ^*∗∗*^*p* < 0.01; and ^*∗∗∗*^*p* < 0.001 (vs. control); ^##^*p* < 0.01; ^###^*p* < 0.001 (vs. Gy).

It has been known that cancer stem cells (also named cancer-initiating cells or cancer stem-like cells) play a central role in tumor progression, metastasis, recurrence, and chemotherapy resistance. Herein, we also detect the effect of chidamide on lung cancer stemness. Results of sphere formation assay demonstrated that the sizes and number of spheres were suppressed by chidamide alone or a combination treatment of chidamide and radiation (Figures 2(a)–2(j)). To determine the population of cancer stem cells, ALDEFLUOR assay was performed. The results demonstrated that chidamide alone or a combination treatment of chidamide and radiation reduced the population of ALDH^+^ cells (Figures 2(k)–2(m)).

### 3.2. Chidamide Alone or a Combinational Treatment with Chidamide and Radiation Upregulates miR-375 Expression

**Figure 3:**
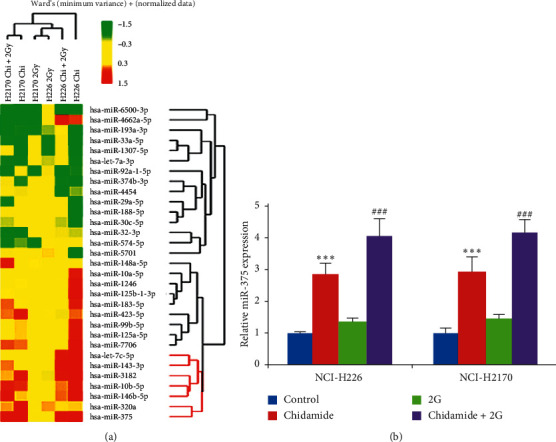
Chidamide alone or chidamide and radiation combinational treatment upregulates miR-375 in NCI-H2170 and NCI-H226 cells. (a) miRNA microarray to identify the differential expressed miRNAs in NCI-H2170 and NCI-H226 cells subjected to radiation (2 Gy), chidamide (300 nM), and their combinational treatment. (b) Real-time PCR to determine the expression of miR-375 in NCI-H2170 and NCI-H226 cells with indicated treatment. Statistical significance was determined by Student's *t*-test and indicated by ^*∗*^*p* < 0.05; ^*∗∗*^*p* < 0.01; and ^*∗∗∗*^*p* < 0.001 (vs. control); ^##^*p* < 0.01; ^###^*p* < 0.001 (vs. Gy).

Next, we sought to elucidate the molecular mechanism underlying antitumor activity of chidamide. As miRNAs play a critical role in tumorigenesis, we hence focused on the expression profile alterations of miRNAs. The Affymetrix miRNA 2.0 Array was applied to identify differentially expressed miRNAs in NCI-H2170 and NCI-H226 cells in response to chidamide, radiotherapy, and their combinational treatment. Among the upregulated miRNAs, miR-375 showed the most remarkable fold (Figure 3(a)). Moreover, real-time PCR results also validated that both chidamide and chidamide plus radiation combinational treatment elevated the expression of miR-375 in NCI-H2170 and NCI-H226 cells (Figure 3(b)). Interestingly, radiation alone could not upregulate miR-375 expression, which indicates a crucial role of miR-375 in mediating the antitumor activity of chidamide against LSCC.

### 3.3. Suppression of miR-375 Reverses the Promoting Effect of Chidamide on LSCC Cell Apoptosis and Attenuates Reduction of Cancer Stem Cells Caused by Chidamide

**Figure 4:**
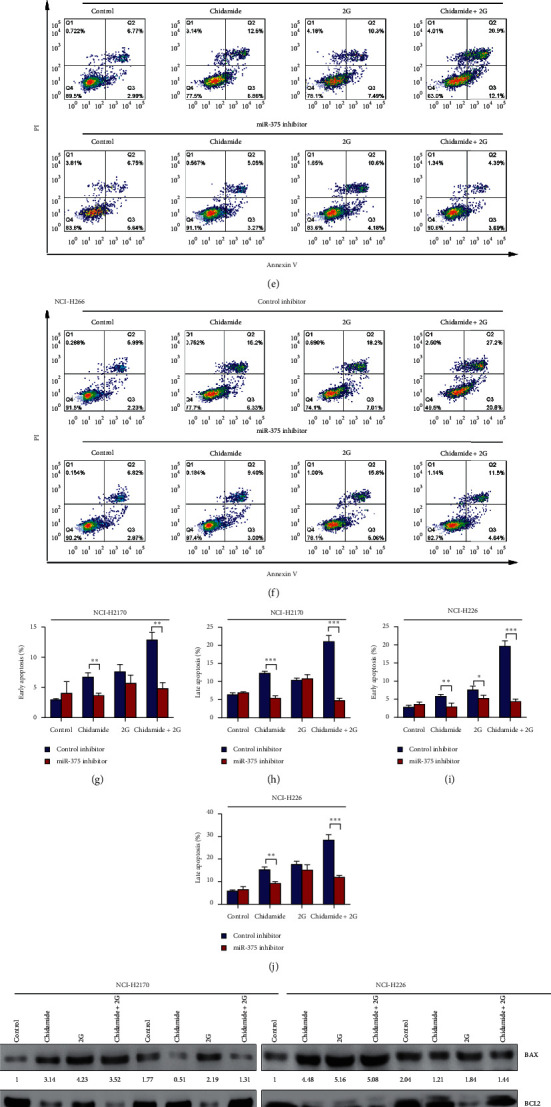
Suppression of miR-375 reversed chidamide and chidamide plus radiation combinational treatment-induced apoptosis in NCI-H2170 and NCI-H226 cells. (a) Real-time PCR to determine the expression of miR-375 in control mimic and miR-375 mimic-transfected NCI-H2170 and NCI-H226 cells with radiation (2 Gy), chidamide (300 nM), and their combinational treatment, respectively. (b) CCK8 assay to evaluate the cell viability of NCI-H2170 and NCI-H226 cells with indicated treatment. (c–j) Annexin V-PI double staining to determine the apoptosis rate of NCI-H2170 and NCI-H226 cells with indicated treatment. (k) The expression of proapoptotic protein BAX and antiapoptotic protein BCL2 in NCI-H2170 and NCI-H226 cells with indicated treatment, determined by western blot analysis. All the data were statistically analyzed by Student's *t*-test. ^*∗*^*p* < 0.05; ^*∗∗*^*p* < 0.01; and ^*∗∗∗*^*p* < 0.001.

**Figure 5:**
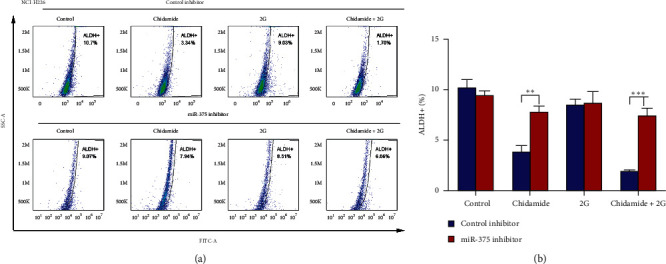
Inhibition of miR-375 attenuated chidamide and chidamide plus radiation combinational treatment-induced suppression of cancer stemness in NCI-H2170 and NCI-H226 cells. (a) ALDEFLUOR assay was performed to evaluate the population of ALDH^+^ cells of NCI-H2170 and NCI-H226 cells with indicated treatment and the representative images were shown. (b) Statistic analysis of ALDH^+^ populations of NCI-H2170 and NCI-H226 cells with indicated treatment. All the data were statistically analyzed by Student's *t*-test. ^*∗*^*p* < 0.05; ^*∗∗*^*p* < 0.01; and ^*∗∗∗*^*p* < 0.001.

To delineate the role of miR-375 in the chidamide-induced LSCC cell apoptosis, we transfected NCI-H2170 and NCI-H226 cells with control inhibitor and miR-375 inhibitor following chidamide, radiation, and their combinational treatment, respectively. CCK8 assay was performed to measure the proliferation rate in these treated cells. As shown in Figures 4(a) and 4(b), transfection of miR-375 inhibitor effectively suppressed the expression of miR-375 both in NCI-H2170 (Figure 4(a)) and NCI-H226 (Figure 4(b)) cells. Moreover, miR-375 inhibition significantly reelevated the proliferation rates of NCI-H2170 and NCI-H226 cells, which were suppressed by chidamide or chidamide plus radiation combinational treatment (Figures 4(c) and 4(d)). In addition, both the chidamide and chidamide plus radiation combinational treatment-induced cell apoptosis were rescued by miR-375 inhibitor (Figures 4(e)–4(j)). To further address the underlying mechanism, western blot analyses were performed to detect the expression of BAX and BCL2 in these treated cells. Data from the western blot analysis revealed that both chidamide and chidamide plus radiation combinational treatment upregulated the protein expression of BAX and downregulated BCL2 protein level, which were diminished by the transfection of miR-375 inhibitor (Figure 4(k)). Moreover, inhibition of miR-375 also reversed the population of ALDH^+^ cells suppressed by chidamide alone or chidamide and radiation combinational treatment (Figures 5(a) and 5(b)).

### 3.4. Chidamide Reduces Xenograft Growth by Elevating miR-375 Expression *In Vivo*

**Figure 6:**
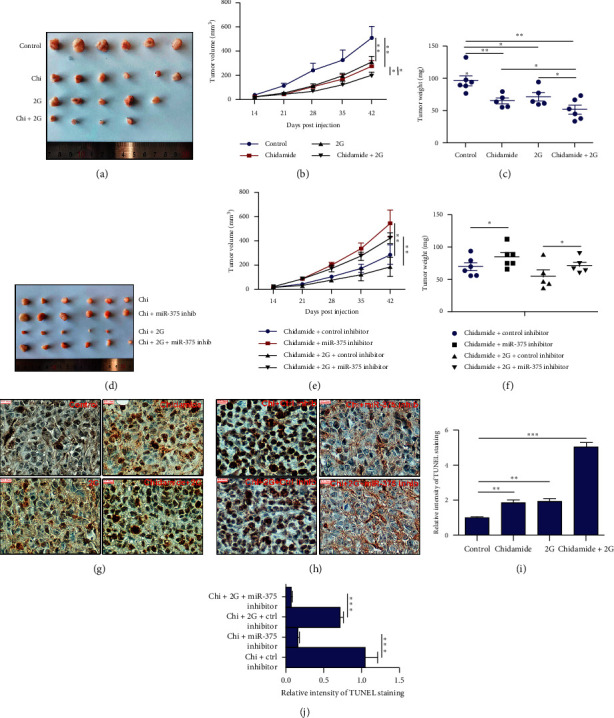
miR-375 inhibition rescued chidamide and chidamide plus radiation combinational treatment-induced tumor growth arrest in NCI-H2170 and NCI-H226 cells, respectively. (a–c) Chidamide, radiation, and their combinational treatment inhibited tumor growth in NCI-H2170 cells. Tumor growth curves and tumor weight analysis are shown in Figures 3(b) and 3(c), respectively. (d–f) Suppression of miR-375 reversed chidamide and chidamide plus radiation combinational treatment-induced tumor growth arrest in NCI-H2170 and NCI-H226 cells, respectively. Tumor growth curves and tumor weight analysis are shown in Figures 3(e) and 3(f), respectively. (g-h) *TUNEL* staining to demonstrate the cell apoptosis rate in indicated xenograft tissues. (i-j) The relative intensity of TUNEL staining in (g) and (h). Scale bars: 100 *μ*m. All the data were statistically analyzed by Student's *t*-test. ^*∗*^*p* < 0.05; ^*∗∗*^*p* < 0.01.

Our *in vitro* results demonstrated that chidamide inhibited LSCC cell proliferation and induced cell apoptosis via upregulation of miR-375 expression. Here we sought to further investigate the *in vivo* antitumor activity of chidamide. Xenograft nude mice were established by subcutaneous inoculation of treated NCI-H2170 cells. The results indicated that the cells treated with chidamide or chidamide plus radiation generated smaller tumors than control (Figures 6(a)–6(c)). Meanwhile, as Figures 6(d)–6(f) show, transfection with miR-375 reelevated the tumor growth rate suppressed by chidamide or chidamide plus radiation combinational treatment. In addition, these results were confirmed by *TUNEL* staining (Figures 6(g)–6(j)).

### 3.5. EIF4G3 Is a Direct Target of miR-375

**Figure 7:**
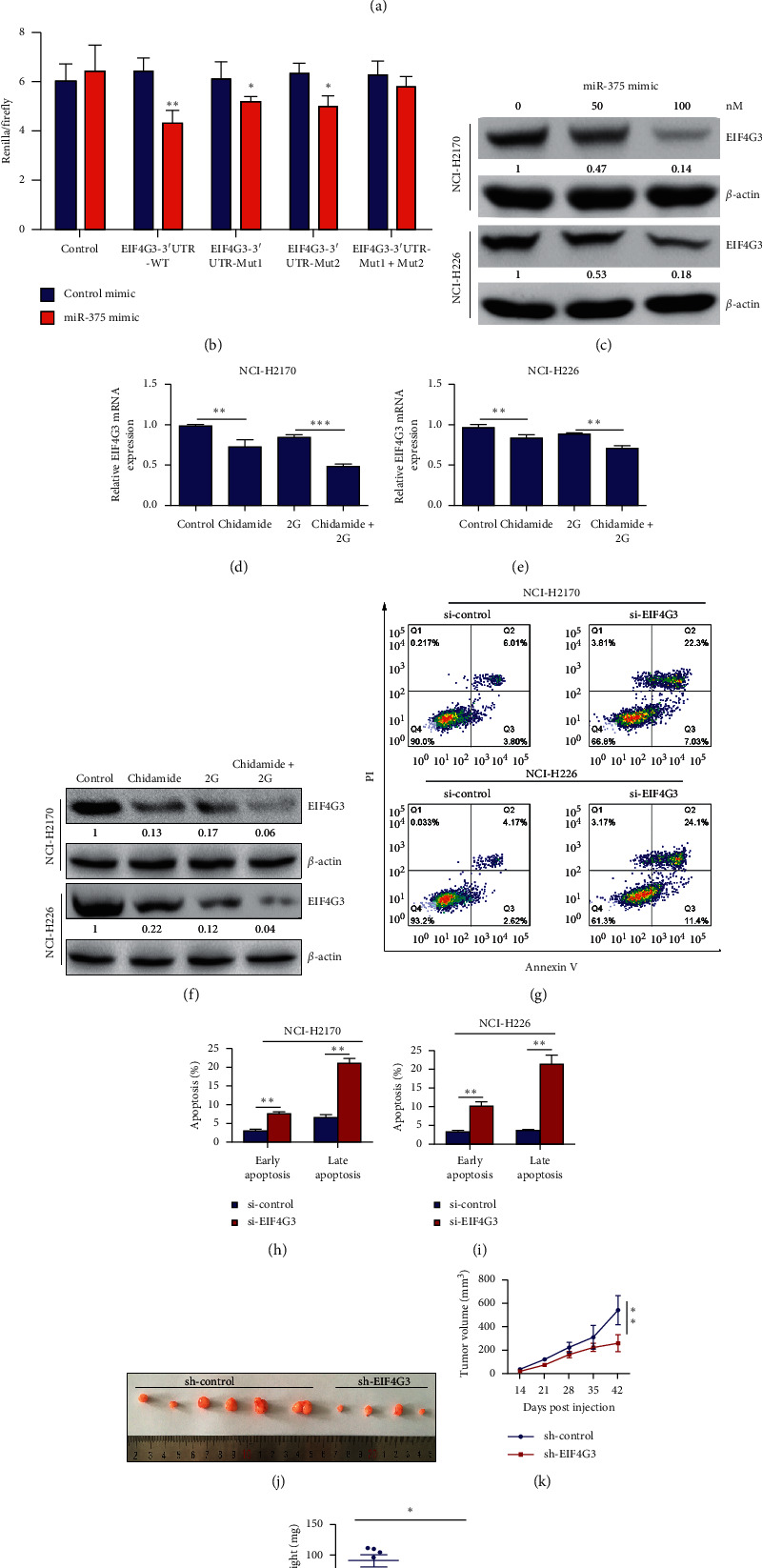
EIF4G3 is a direct target of miR-375. (a) Schematic view to present putative miR-375 binding sites in the 3′UTR region of EIF4G3. The mutant sequences are shown below. (b) Luciferase reporter assay to assess the effect of miR-375 on the transcription of Renilla luciferase with 3´UTR of EIF4G3 expression. Data were shown as means ± SEM. Each group was performed in six biological replicates. ^*∗*^*p* < 0.05; ^*∗∗*^*p* < 0.01. (c) Western blot analysis to determine the protein expression of EIF4G3 in NCI-H2170 and NCI-H226 cells transfected with miR-375 mimic at the concentration of 0 nM, 50 nM, and 100 nM, respectively. (d)-(e) Detection of the mRNA expression of EIF4G3 in NCI-H2170 and NCI-H226 cells with indicated treatment. (f) Western blot analysis to detect the expression of EIF4G3 in the indicated groups. (g–i) The apoptosis rate of NCI-H2170 and NCI-H226 cells transfected with 100 nM of EIF4G3 siRNA, compared with control. (j–l) EIF4G3 inhibition significantly suppresses tumor growth *in vivo* in NCI-H2170 cells. Tumor growth curves and tumor weight analysis are shown in Figures 5(h) and 5(i), respectively. All the data were statistically analyzed by Student's *t*-test. ^*∗*^*p* < 0.05; ^*∗∗*^*p* < 0.01; and ^*∗∗∗*^*p* < 0.001.

Furthermore, we identified the target genes of miR-375 using TargetScan online software (http://www.targetscan.org/vert_72/). Among the numerous targets, eukaryotic translation initiation factor 4 gamma 3 (EIF4G3) was selected as the candidate target gene for miR-375 for further analysis, as its 3′-UTR contains two conserved binding regions of miR-375. The binding sites between miRNA-375 and EIF4G3 are shown in Figure 7(a). To clarify whether EIF4G3 is a direct target of miR-375, a dual-luciferase reporter assay was applied to determine the luciferase activities of EIF4G3 3′-UTR. As shown in Figure 7(b), transfection of miR-375 mimic significantly reduced the luciferase activity of the wild-type 3´-UTR of EIF4G3 compared with the control mimic-transfected cells (Figure 7(b)). However, less significant differences were found between cells transfected with control mimic and the miR-375 mimic when cotransfected with the mutated 3′-UTR of EIF4G3 (Figure 7(b)). Additionally, results of western blotting revealed that miRNA-375 significantly downregulated EIF4G3 (Figure 7(c)).

### 3.6. Silence of EIF4G3 Promotes Cell Apoptosis and Suppresses Xenograft Growth in NCI-H2170 and NCI-H226 Cells

To determine the role of EIF4G3 in the biological behaviors of LSCC cells, we first detected the expression of EIF4G3 in NCI-H2170 and NCI-H226 cells treated with chidamide or chidamide plus radiation combinational treatment. The results demonstrated that both chidamide and chidamide plus radiation combinational treatment suppressed EIF4G3 expression both at the mRNA and protein levels (Figures 7(d)–7(f)). Next, we silenced the expression of EIF4G3 in NCI-H2170 and NCI-H226 cells and found that silence of EIF4G3 significantly induced cell apoptosis (Figures 7(g)–7(i)). Moreover, silence of EIF4G3 in NCI-H2170 cells obviously suppressed xenograft growth (Figures 7(j)–7(l)).

Taken together, our study systematically demonstrates that chidamide and radiation synergistically promote LSCC cell apoptosis and suppressed tumor growth and stemness by modulating the miR-375-EIF4G3 axis.

## 4. Discussion

The HDAC inhibitors can be used for various diseases, some of which have entered clinical trials. In cancer, HDAC inhibitors are becoming promising novel tumor therapeutic drugs exerting anticancer function across a wide range of cancers, especially in leukemia. Chidamide, an orally active novel HDAC inhibitor of the benzamide class, selectively inhibits HDAC1, HDAC2, HDAC3, as well as HDAC10. In pancreatic cancers, chidamide augments gemcitabine-induced cell growth arrest and apoptosis by downregulating the antiapoptotic gene MCL-1 [31]. Chidamide has been tested extensively for its tumor inhibitory activity. In colon cancer cells, chidamide suppresses cell proliferation and induces cell cycle arrest by inhibiting the PI3K/AKT and RAS/MAPK signaling pathways. In non-small-cell lung cancer cell lines, chidamide and carboplatin synergistically induce cell growth arrest [11]. In the present study, we demonstrated that chidamide-induced cellular growth inhibition of NCI-H2170 and NCI-H226 LSCC cells and it synergistically augmented radiation-induced cell apoptosis. Previous reported studies have demonstrated that HDAC inhibitors show significant single-agent anticancer activity in T-cell lymphomas. Consistent with these results, our data also show that chidamide alone induces cell apoptosis and suppresses tumor growth in LSCC.

Although previously published data also demonstrate chidamide induces cell apoptosis, the underlying mechanism is not very clear. Our data showed that both chidamide and chidamide plus radiation combinational treatment induced the expression of endogenous miR-375 in NCI-H2170 and NCI-H226 cells. Moreover, rescue experiments indicated that the apoptosis-promoting function of chidamide depended on the upregulation of miR-375 expression. In addition, the scaffold protein EIF4G3 was identified as a direct target of miR-375 and the suppression of EIF4G3 induced cell apoptosis and inhibited tumor growth in NCI-2170 and NCI-H226 cells. These data conclude that chidamide exhibits a synergistic effect with radiation therapy on LSCC by modulating the miR-375-EIF4G3 axis. Our finding may represent a universal mechanism underlying the synergistic antitumor interactions between HDAC inhibitors and DNA damaging agents in tumorigenesis, which should be confirmed in our future studies.

In the study of NCI-H2170 and NCI-H226 cell lines, it has been found that chidamide, radiation, and their combinational treatment could exert an anti-LSCC effect. However, unlike chidamide and chidamide plus radiation combinational treatment, radiation alone could not upregulate the expression of miR-375 and downregulate the expression of EIF4G3. These results suggest the miR-375/EIF4G3 axis may not be involved in the regulation of radiotherapy alone-induced cell apoptosis. On the other hand, acquired drug resistance frequently occurred to destroy effective therapy with chemotherapeutic agents, leading to an unsatisfactory clinical outcome. Therefore, HDAC inhibitor combined with radiation at different dose might have multiple targets and pathways to induce cell apoptosis.

In conclusion, our results systematically explored the role of miR-375/EIF4G3 axis in chidamide-induced LSCC apoptosis and tumor growth arrest, which may afford an effective strategy to overcome the drug resistance of chidamide in clinical cancer therapy.

## Data Availability

The data used to support the findings of this study are available from the corresponding author upon request.

## Abbreviations

miRNA: MicroRNA
HDAC: Histone deacetylase
PTCL: Peripheral T-cell lymphoma
LSCC: Lung squamous cell carcinomas
SCLC: Small-cell lung cancer
NSCLC: Non-small-cell lung cancer
LCLC: Lung large-cell carcinoma
LungNET: Lung neuroendocrine tumor
LUAD: Lung adenocarcinoma
3′-UTRs: Untranslated regions
EIF4G3: Eukaryotic translation initiation factor 4 gamma 3
FBS: Fetal bovine serum
ATCC: American Type Culture Collection
DMEM: Dulbecco's Modified Eagle Medium
DMSO: Dimethyl sulfoxide.
